# A clinical-translational review of sleep problems in neurodevelopmental disabilities

**DOI:** 10.1186/s11689-024-09559-4

**Published:** 2024-07-20

**Authors:** Sarika U. Peters, Althea Robinson Shelton, Beth A. Malow, Jeffrey L. Neul

**Affiliations:** 1 Department of Pediatrics, Vanderbilt University Medical Center, Nashville, USA; 2 Vanderbilt Kennedy Center for Research on Human Development, One Magnolia Circle, Room 404B, Nashville, TN 37203 USA; 3 Department of Neurology, Vanderbilt University Medical Center, Nashville, USA

**Keywords:** Sleep, Neurodevelopmental disabilities, Animal models, Rett syndrome, Angelman syndrome, Down syndrome, Autism

## Abstract

Sleep disorders are very common across neurodevelopmental disorders and place a large burden on affected children, adolescents, and their families. Sleep disturbances seem to involve a complex interplay of genetic, neurobiological, and medical/environmental factors in neurodevelopmental disorders. In this review, we discuss animal models of sleep problems and characterize their presence in two single gene disorders, Rett Syndrome, and Angelman Syndrome and two more commonly occurring neurodevelopmental disorders, Down Syndrome, and autism spectrum disorders. We then discuss strategies for novel methods of assessment using wearable sensors more broadly for neurodevelopmental disorders in general, including the importance of analytical validation. An increased understanding of the mechanistic contributions and potential biomarkers of disordered sleep may offer quantifiable targets for interventions that improve overall quality of life for affected individuals and their families.

## Background

Sleep disorders are extremely common in neurodevelopmental disabilities (NDD) and occur at a significantly higher rate as compared to typically developing children, adolescents, and adults [1]. Some studies show that between 50 and 95% of individuals with NDDs exhibit sleep problems [2], with sleep disturbances often emerging from a very young age and being prevalent throughout the lifespan [1]. Sleep problems are one of the major co-occurring conditions in many NDDs [2], place a large burden on children and families, and are associated with a range of cognitive and behavioral phenotypes [1, 3]. In fact, sleep problems are often included at least as a secondary outcome measure as part of clinical trials in a variety of single gene disorders and/or as a core feature to consider as part of Clinical Global Impression Severity Scales (CGI-S) [4, 5]. Numerous studies reveal that child sleep disturbances in NDDs adversely impact family quality of life [6–8]. Sleep problems in NDDs are not always related to behavioral sleep hygiene. Indeed, studies suggest mechanistic contributions to sleep disorders in NDDs, and investigations of sleep disturbances in animal models of these single gene disorders can offer potential clues as to etiology [9], as well as clinical translational approaches to treatment and mitigation. In this review article, we discuss the prevalence and etiology of sleep disturbances in two single gene disorders, Rett Syndrome, and Angelman Syndrome. We then expand this discussion to include more commonly occurring NDDs, Down Syndrome, and autism spectrum disorders and then discuss strategies for novel methods of assessment more broadly for NDDs in general. Increased understanding of the mechanistic contributions and potential biomarkers of disordered sleep in these NDDs may offer quantifiable targets for interventions that improve overall quality of life for affected individuals and their families. Specific sleep problems in these NDDs can include insomnia, sleep-disordered breathing, disruption to circadian rhythms, parasomnias, sleep-related movement disorder, and/or excessive daytime sleepiness. These sleep problems occur to varying degrees across the different disorders presented here and will be discussed in more detail within the sections for each disorder. Insomnia is defined as persistent difficulty with sleep initiation, duration, consolidation, or quality that occurs despite adequate opportunity and circumstances for sleep and results in daytime impairment [10]. Sleep-disordered breathing (SDB) includes a range of respiratory disorders such as snoring, sleep-induced hypoxemia, sleep-related hypoventilation, upper airway resistance syndrome, obstructive sleep apnea (OSA), and/or central sleep apnea [10]. Parasomnias include sleep terrors, sleepwalking, and confusional arousals and can occur during entry into sleep, during sleep, or during an arousal from sleep. Sleep-related movement disorders are movements that impact sleep onset or duration and can include bruxism, restless leg syndrome, and restless sleep disorder [11]. Disorders of hypersomnolence (narcolepsy) present with excessive daytime sleepiness. An overview of these sleep problems investigated in clinical studies as well as potential underlying etiology as investigated within specific genetic strains of animal models within these NDDs are presented within this review.

Sleep is regulated both by a homeostatic (Process S) and a circadian process (Process C) [12]. Together these two processes determine most aspects of sleep and related variables like sleepiness and alertness. Sleep homeostasis refers to the notion that when there is a loss of sleep, it elicits a compensatory increase/drive in the intensity and duration of sleep. The homeostatic mechanism regulates sleep intensity and the depth and maintenance of sleep, while the circadian clock regulates the timing of sleep. In each of the NDDs discussed in the review, studies from animal models suggest that the underlying etiology of sleep problems may include homeostatic and/or circadian processes. Circadian rhythms can be investigated by exposing mice to light/dark cycles, and sleep homeostasis is usually investigated by actively preventing the animal from engaging in sleep (despite the desire/drive to sleep). The “pacemaker” of the circadian system is located in neurons within the suprachiasmatic nucleus (SCN) of the hypothalamus, and examination of disruptions to homeostasis and circadian rhythms within neurons in the SCN has provided insights into potential mechanisms. The SCN controls the timing of the sleep-wake cycle and coordinates circadian changes in activity across the brain and body tissues.

### Rett Syndrome

Rett Syndrome (RTT, MIM 312,750) is a severe neurodevelopmental disorder that primarily affects females. The prevalence is thought to be around 1:10,000 female births (total cases in US ~ 10,000). RTT is caused by mutations in the methyl CpG-binding protein 2 gene (*MECP2*) [13] and is associated with loss of function of MeCP2. The typical/classic form of RTT is characterized by regression, loss of purposeful hand skills and replacement with stereotyped hand movements/hand-washing motions, limited speech, dyspraxia, and abnormal muscle tone [14]. Included in the RTT phenotype are a variety of symptoms suggestive of autonomic dysregulation including breathing irregularities (e.g. hyperventilation, apnea, breath holding), heart rate variability, and temperature dysregulation [15–17]. Associated features include impaired sleep, breathing disturbances, bruxism, vasomotor disturbances, abnormal muscle tone, diminished response to pain, and scoliosis, among others [14, 17]. Sleep problems are highly prevalent in RTT [18–20] and are part of the supportive clinical criteria [14] with around 80% of the population being affected. Recent studies show that sleep difficulties, autonomic dysfunction, and milder clinical severity are associated with higher levels of anxiety in RTT [21, 22], suggesting adverse impacts of sleep problems on mental health as well as physical health. Disrupted sleep has a large burden on the health and well-being of both the child and caregivers affected by RTT, and clinical management is symptomatic, and does not appear to be associated with clear improvements [20].

#### Sleep disturbances in clinical research studies in RTT

Impairments in various aspects of sleep are quite common in RTT, with a recent study showing that 79–85% of caregivers reported their children with RTT experienced at least one sleep problem, including frequent nighttime waking, screaming spells and/or laughing at night (parasomnia), sleep-related movement (e.g., bruxism, restless legs), or daytime sleepiness [20]. Studies utilizing polysomnography (PSG) also show that individuals with RTT have increased sleep latency, increased wake after sleep onset (WASO), and reduced sleep efficiency [23, 24]. A case series of *n* = 13 participants with RTT also using PSG showed increased limb movements during sleep and also showed REM sleep is attenuated in RTT [25]. Breathing disturbances while awake have been described as alternating hyperpnea followed by apnea [26], which can be associated with cyanosis. Findings regarding SDB in RTT have been contradictory. Although earlier studies suggested that the breathing disturbances are normalized during sleep [26], more recent studies utilizing PSG show that both OSA and central sleep apnea (associated with hypoventilation) are common [24, 27] in RTT. A case series of *n* = 11 children with RTT [24] showed that 54.5% had OSA in both non rapid eye-movement (NREM) and rapid eye movement (REM) sleep which was unrelated to their clinical features. This study also revealed hypoxemia throughout nocturnal sleep in RTT [24]. Delta power on sleep electroencephalography (EEG) is considered a biomarker of sleep homeostasis since it is associated with sleep intensity and duration. Results from a sleep EEG study in RTT showed an increase in delta power during slow wave sleep (SWS) and decreased time spent in SWS in 2-9-year-old girls with RTT [28]. While delta power usually decreases over consecutive slow wave cycles during a night of sleep, this pattern was not noted in RTT [28], and is suggestive of chronice sleep deprivation [29]. In sum, sleep problems affect a majority of individuals with RTT, are associated with adverse outcomes in terms of mental and physical health, adversely impact caregiver quality of life, and the most recent studies suggest that sleep disruption, autonomic dysfunction, and anxiety are interconnected in RTT [30] and must be considered in tandem.

#### Animal models of sleep problems in RTT

The discovery of the X-linked *MECP2* gene, which encodes the transcriptional regulator methyl-CpG-binding protein 2 (MeCP2), as a primary cause of RTT allowed for the creation of animal models to study underlying pathology and develop new treatments [13]. The first models of RTT were male mice with a total knockout of the *MECP2* gene and complete disruption of the MeCP2 protein [31, 32] although there since have been other genetic models that model human point mutations, and a truncation mutation [33]. These mouse models recapitulate many of the features of Rett syndrome including stereotypic forelimb motions, uncoordinated gait, reduced spontaneous movement, and irregular breathing. These animal models serve to cross-validate findings from human clinical research studies and vice-versa. Sleep changes can be assessed in mice by investigating their activity in cages equipped with running wheels and by obtaining EEG recordings during the day/night cycle (assessing circadian rhythms and homeostasis). In one study, the investigators examined whether the circadian system was disrupted in Mecp2^−/y^ mice and found a disruption to the circadian clock within the SCN such that mutant mice exhibited a decreased strength and precision of daily circadian rhythms and fragmented sleep [34]. Another study using a different genetic strain, the Mecp2^tm1.1Bird^ mouse, found a significantly enhanced waking state and shorter duration of REM sleep, increased sleep fragmentation, and increased sleep inertia that recapitulate sleep problems described in RTT patients [35]. The possible mechanistic underpinnings of the sleep-wake cycle in RTT might be attributable to MeCP2 binding and transcriptionally activating the circadian clock genes, Per1 and Per2 [36], and that MeCP2 protein is highly expressed in the suprachiasmatic nucleus (SCN) [37]. More specifically, findings from the Mecp2^−/y^ model showed that there was a reduction of neurons in the SCN expressing vasoactive intestinal peptide (VIP) as well as reduced spontaneous neural activity [34]. Circadian disruption was noted in the SCN and in peripheral organs, indicating a general disorganization of the circadian system. Taken together, findings from studies of animal models suggest a role for MeCP2 in the circadian timing system and provide a possible mechanistic explanation for the sleep/wake disturbances observed in RTT patients. It will be important, however, to determine the consistency of these findings across different genetic strains of mice. In sum, although studies in humans and animal models have contributed to a broader understanding of potential underlying mechanisms of sleep disturbances in RTT, further research is needed to investigate the molecular, neuronal, and non-neuronal pathways underlying sleep disorders.

### Angelman syndrome

Angelman syndrome (AS) is a neurodevelopmental disorder affecting both females and males, with an estimated prevalence of around 1:15,000. AS is caused by the loss of function of the maternally expressed Ubiquitin-protein ligase E3A (*UBE3A*). Most cases (70%) of AS are caused by a deletion in the maternal copy of chromosome 15q11.2-q13. Other forms of AS are attributable to paternal uniparental disomy (3% of cases), an imprinting center defect (6% of cases), a mutation in the maternally inherited allele of *UBE3A* (11% of cases) [38]. The phenotype, present from birth, is characterized by absent or minimal spoken language, gait abnormalities, epilepsy, a happy/excitable personality, and abnormal movements [39]. Features of autism are also prominent within AS [40, 41]. Although phenotypic features vary depending upon the molecular subtype of AS [42], sleep problems are present in up to 80% of individuals with AS [43] and have not differed by molecular subtype [44]. Within the consensus guidelines for AS, abnormal sleep-wake cycles and diminished need for sleep are considered associated features [43]. Sleep problems are a major contributor to parental/caregiver stress in AS [45–47].

#### Sleep disturbances in clinical research studies in AS

Most clinical research studies of sleep problems in AS have involved parent/caregiver questionnaires, although some more recent studies have utilized PSG and actigraphy. Although sleep problems seem to be worse in younger children, being most prevalent between the ages of 2–9 years [48, 49], several studies suggest that a significant percentage of individuals with AS exhibit sleep problems that persist into adolescence and adulthood. Sleep problems are pervasive enough and adversely impact caregiver quality of life enough in AS to be included in a clinician-reported and a caregiver-reported outcome assessment scale that is being utilized in current clinical trials [50]. Seizures are very common in AS, and a high percentage of those with AS who have epilepsy (79% in one study) also have sleep problems, with the severity of seizures correlating with sleep disturbances [51]. Seizures disrupt sleep architecture, fragment sleep, and can decrease REM sleep [49]. Results from questionnaire studies show that between 35 and 60% of individuals with AS have difficulty initiating sleep and/or maintaining sleep [48, 52, 53]. Night wakings are very common in AS and may be accompanied by behaviors such as screaming [48]. Other salient findings from sleep questionnaire studies in AS include snoring, sleep terrors, sleepwalking, sleep fragmentation, nocturnal hyperkinesia, nocturnal laughing, and a reduced need for sleep [46, 48, 52, 53]. Some reports also suggest that individuals with AS only sleep 5–6 h/night and do not have daytime sleepiness even with fragmented and/or reduced night-time sleep [52]. PSG is very challenging in AS, and, as such, studies to date consist of smaller sample sizes (e.g., *n* = 10). Results from PSG studies suggest increased sleep latency due to difficulties with settling, decreased total sleep time, reduced sleep efficiency, and reduced percentage of slow wave sleep [54, 55]. In addition, these studies showed that the percentage and duration of REM sleep was significantly lower, and the percentage of slow wave sleep was significantly higher [54, 55]. Sleep EEG coherence is a measure of the brain’s connectivity during sleep, and it is assessed by examining interactions between adjacent brain regions (short-range) and between more distant regions (long-range). Gamma band coherence is usually lower during sleep, and increases suggest attentive wakefulness and tend to occur during REM sleep. In AS, a recent retrospective study of *n* = 28 children (ages 4–11 years) found *increased* long-range EEG coherence in the gamma band during sleep and decreased sleep spindle number and duration [56] which could have a role in neuronal plasticity as well as implications for memory and learning [57, 58]. To summarize, sleep problems are pervasive in AS and are often associated with epilepsy and increased behavioral challenges and adversely impact quality of life for affected individuals and their caregivers.

#### Animal models of sleep problems in AS

The most commonly used mouse model of AS recapitulates many phenotypic features observed in AS patients [38] including epilepsy, motor deficits, abnormal EEG, abnormal sleep patterns, increased anxiety, and repetitive behaviors. This model has been valuable for understanding disease processes in AS and in identifying appropriate drug targets [59]. Two studies in AS mice evaluated changes in the sleep-wake cycle [60, 61]. In one study [61], they found that circadian rhythms are intact, but abnormal sleep patterns arise from a deficit in accumulation of sleep drive (i.e. disruption to sleep homeostasis). In this study, they also determined that Ube3a protein was present in many neurons of the SCN, suggesting that it acts as a novel genetic regulator of sleep homeostasis. In contrast, in a different study Shi et al., 2015 showed alterations to circadian rhythms where they showed a longer circadian period that leads to delayed phase [60], which they hypothesize could account for the difficulty with settling to sleep (sleep onset latency) and shorter sleep duration in individuals with AS. These different findings suggest the importance of continuing to investigate how *UBE3A* affects sleep patterns in animal models, and differences in genetic backgrounds/strains could account for these differences. Although there are still gaps in understanding the mechanistic basis of sleep problems in AS, it is important to note that in deletion forms of AS, there is a disruption in the gene that encodes for the B_3_ subunit of the gamma-aminobutyric acid–mediated (GABA) receptor, and alterations to this receptor may cause inhibitory influences on thalamocortical interactions that could be responsible for the sleep problems in AS [49]. In homozygous Gabrb3-knockout mice (one of the models used to assist with studying AS), studies of reciprocal inhibitory connections demonstrated abolition of GABA-mediated inhibition in the thalamic reticular nucleus as well as an increase in oscillatory synchrony [62]. Excitingly, recent therapeutic advances in AS pre-clinical studies show that treatment with an antisense oligonucleotide (ASO) rescues abnormal EEG rhythms and sleep disturbances [63]. Since these compounds have started to advance to human clinical trials, it will be important to determine whether some of the sleep disturbances in AS are normalized in human clinical studies as well. In sum, sleep is significantly disrupted in AS but contributions to sleep disruption are likely multi-faceted and require further study to determine the underlying pathophysiology. Despite this, recent advances in therapeutic development suggest that normalization of at least some of the sleep disturbances could be possible with novel compounds.

### Down syndrome

Down Syndrome (DS) is the most common genetic cause of NDD but is very complex genetically, with potentially more than 500 genes that could be overexpressed on chromosome 21. There are several significant medical co-occurring conditions in DS including: congenital heart disease, hypothyroidism, feeding problems and gastrointestinal issues [64], and obesity. Obesity in DS starts to become a concern between the ages of 4–5 years [65, 66], and this is the age when parents also begin to become increasingly concerned about challenging behaviors [67]. Individuals with obesity and DS are more likely to also have obstructive sleep apnea (OSA) [66].

#### Sleep disturbances in clinical research studies in DS

Studies that have utilized parental questionnaires and those that have conducted patient EEG recordings consistently show sleep architecture to be altered in children in DS with alterations consisting of reduced time spent in NREM sleep, increased sleep latency, and increased night wakings [68, 69]. A study utilizing actigraphy, sleep diaries, and PSG showed that children with DS were averaging 6.9 to 7.9 h of sleep, with poor sleep efficiency [70]. Studies have suggested 50 to 79% of children with Down syndrome have OSA compared to 1–4% of the rest of the population [71]. Sleep problems in DS can start from a very young age but are prevalent throughout the lifespan with studies also showing that 78 to 90% of adults with DS have OSA [72]. Increased prevalence of OSA in DS could be attributable to anatomic differences such as hypotonia, macroglossia, and midface hypoplasia [71]. In addition, many children with DS have enlarged adenoids and tonsils, which also cause obstruction within the already narrowed airway [73]. Importantly, while adenotonsillectomy (AT) is considered first-line therapy for the treatment of pediatric OSA, persistent OSA after AT occurs in many (58%) with DS [74, 75] requiring more intensive follow-up. OSA in DS is associated with a range of medical, cognitive, and behavioral outcomes such as lower verbal IQ [76], Attention-Deficit/Hyperactivity Disorder (ADHD) [77–79], and impaired autonomic/cardiovascular control [80]. A recent study showed that children with DS had elevated heart rate during N2 (NREM) and N3 (NREM) sleep as compared to typically developing children and did not exhibit the typical fall in heart rate from wake to sleep [80]. A study in middle-aged adults with DS shows that more disrupted sleep is associated with lower white matter integrity, increasing the risk of Alzheimer’s disease [81]. Individuals with DS frequently exhibit an abnormal PSG, with high sleep fragmentation, manifested by frequent awakenings and arousals, considerable limb movement [82], and frequent OSA [83]. Children with DS often require repeated PSG because of continued difficulties with sleep problems (e.g., even after AT) and/or a resurgence of sleep problems during adolescence [71] that coincides with puberty and the onset of obesity. In sum, sleep problems are extremely prevalent in DS, with most individuals having OSA, and this has a significant long-term impact on overall functioning, as well as the quality of life for individuals with DS as well as their families.

#### Animal models of sleep problems in DS

In contrast to RTT and AS, the development of animal models to study the underlying pathophysiology of DS and to develop and test potential therapies has been more challenging, in part because no model has captured all of the triplicated region. Animal models that have been developed for DS involve transgenic overexpression of single genes or larger human genomic segments and creation of mouse models carrying a partial triplication in regions of MMU16 syntenic to human chromosome 21 (segmental trisomy models or Ts). Among these models, to date, the Ts65Dn is mostly commonly used in research because it contains triplications of partially overlapping segments in the critical DS region and cover most of the region triplicated in humans [84]. Ts65Dn has also formed the basis of pre-clinical justification for clinical trials [85]. These mice recapitulate some of the phenotype in DS including intellectual disability (ID), hyperactivity, craniofacial malformations, and motor dysfunction [86]. The Tc1 (transchromosomic, Tc(Hsa21)1TybEmcf) line is a trans-species aneuploid line expressing a large portion of Hsa21 (83%, 269 genes) as a third copy. Most studies suggest no alterations in circadian rhythms for Ts65Dn animals. The mice do, however, exhibit sleep fragmentation and have OSA in REM sleep [87]. Tc1 mice exhibit moderate disruptions in rest/activity patterns and hyperactive episodes, with circadian rhythms also appearing similarly unaffected [88]. EEG signals have also been obtained in several mouse models of DS. Findings have differed, depending upon the mouse strain being studied. In one study using the Ts65Dn mouse, investigators found increased waking periods at the expense of non-REM sleep, increased power in theta waves during sleep, and a delayed sleep rebound after sleep deprivation [89]. In contrast, TC1 mice had limited sleep and EEG abnormalities, showing only a delayed sleep rebound after sleep deprivation and no difference in the power of theta oscillations [89]. Unlike RTT and AS, the potential molecular mechanisms underlying sleep problems in DS are less well-understood. Because of the challenges inherent with animal models of DS with regard to translational research, including that a clinical trial was halted in phase 2 because of failure to recapitulate pre-clinical findings from findings with the Ts65DS model, a newer mouse model of DS has been created, the TcMAC21 [90]. These mice have many of the same phenotypic features of DS that are evident in humans, including a distinct facial structure, a greater prevalence for congenital heart defects, a smaller-than-usual cerebellum and learning difficulties [90]. To date, sleep has yet to be studied in this newer model. It will therefore be important to continue to investigate sleep abnormalities in animal models of DS, to develop further treatment and interventions.

### Autism

Autism spectrum disorder (ASD) now affects approximately 1 in 36 children and is around 3.8 times as prevalent among boys as among girls [91]. A small percentage of individuals with ASD have a known genetic etiology, typically in the form of rare variants, while most cases are idiopathic. ASD is characterized by impairments in social communication, accompanied by restricted/repetitive interests and behaviors. ASD is very heterogeneous in terms of phenotypic presentation and functional abilities, especially compared to AS, RTT, and DS. It is important to note, however, that a proportion of individuals with RTT, AS, and DS also meet criteria for ASD, and there are potential overlapping pathways [40, 41, 66, 92, 93]. In addition, those with co-occurring diagnoses of autism often exhibit higher levels of impairment, and one recent study in DS showed that those individuals who had both DS and ASD had more negative effects on sleep architecture. Most of the animal models for ASD to date have therefore been developed using single gene disorders [94]. This approach has allowed for the potential study of shared phenotypes, inclusive of sleep problems and underlying cellular and molecular pathways [95], and the knowledge gleaned from single gene disorders has applicability to ASD.

#### Sleep disturbances in clinical research studies in ASD

Studies of parent-reported sleep problems of ASD reveal prevalence rates ranging between 40 and 80% [1, 96–98]. The most common problems that are reported include difficulties with initiating and maintaining sleep, frequent and prolonged night awakenings, early morning waking, and irregular-sleep wake schedules [99–102]. Reduced total sleep time, increased daytime sleepiness, and night-time laughing and/or talking are also common [97, 99, 100, 102]. Sleep problems for individuals with ASD can persist through adulthood [103], and one study found that adults with ASD had lower sleep efficiency as compared to neurotypical adults. Studies using PSG have shown increased nightwakings, reduced total sleep time, lower sleep efficiency, reductions in REM and non-REM sleep, and longer latency to fall asleep [99, 104]. An increased incidence of sleep problems in those with ASD has been associated with more behavioral difficulties (e.g., aggression, self-injury, anxiety, hyperactivity, irritability, and inattention) [105, 106], increased sensory sensitivity [107, 108], more pronounced repetitive behaviors, and more impairments in social communication [109]. As with the other NDDs discussed here, higher rates of sleep problems in ASD are associated with higher rates of caregiver burden and family stress [105, 110].

#### Etiology of sleep problems in ASD

The underlying etiology of sleep problems in ASD is multifaceted, with possible contributions of neurobiological factors, as well as medical and behavioral factors. Although ASD is genetically heterogeneous, there is emerging evidence that neurotransmitters that regulate sleep and wake cycles such as serotonin, and the hormone, melatonin could contribute to sleep problems in ASD in a subset of individuals. Melatonin is produced by the pineal gland and regulates sleep/wake cycles in humans. Some studies show decreased levels of melatonin in serum, saliva, and/or urine in children and adolescents with ASD [111, 112]. This reduction in melatonin is thought to be associated with a variation in a gene associated with the serotonin-melatonin synthesis pathway, the acetyl serotonin O-methyltransferase (*ASMT*) gene [111, 113]. *ASMT* converts serotonin into melatonin [114] and genetic variants within the promotor region are more frequent in individuals with ASD and are associated with a decrease in the number of ASMT transcripts [111, 115–117], although this impacts a very small subset of individuals. In addition, some studies suggest alterations of the serotonergic signaling system are involved in the pathophysiology of ASD, at least in some individuals [118–120]. One study showed that 40% of individuals with ASD had abnormally high levels of serotonin in blood and 51% had abnormally low levels of melatonin [113], with other work documenting normal overnight blood or evening salivary melatonin levels [103, 121]. These studies consisted of small sample sizes and may not be generalizable given the way samples were collected (in dim light or not). Additionally, studies have shown that supplementation with melatonin can be effective in treating sleep problems in some (53.7% in a recent randomized control trial) [122], but not all individuals with ASD, especially those with night wakings or short sleep duration [103, 123]. Prolonged release melatonin can be helpful for night wakings and short sleep duration, but requires swallowing a pill whole, without it being crushed (to maintain the prolonged release packaging). Given the inconsistency of responses to medications such as melatonin and the heterogeneity within ASD, behavioral strategies are typically recommended as first-line interventions and focus of treatment [100].

### Medical/behavioral/environmental contributions to sleep problems in NDDs

Beyond the neurobiological contributions to sleep problems that have been described for each of the conditions reviewed here (RTT, AS, DS, ASD), there are also potential medical, behavioral, and/or environmental contributions to sleep difficulties. Gastrointestinal issues such as constipation and reflux are very common across all of these NDDs, and many individuals with NDDs are not able to verbally express their discomfort, which can exacerbate sleep problems in particular. For example, constipation can lead to abdominal cramping, which can interfere with a proper night’s sleep. Gastroesophageal reflux may be worsened by lying down at night and could be reflected in an increased latency to falling asleep/bedtime resistance. Sleep dysfunction is also very common in those with NDDs who have epilepsy [124]. Epilepsy disrupts sleep, and sleep disturbances can also lower the seizure threshold [8]. Sleep disruption is also a common side effect of many medications used to treat co-occurring conditions across all of these NDDs (anxiety, irritability, epilepsy, ADHD, etc.). Some medications have properties that contribute to difficulties with sleep onset and/or sleep maintenance. Selective serotonin reuptake inhibitors (SSRIs), for example, can disrupt sleep continuity, and/or decrease total sleep time, increase wake time, and increase stage N1 sleep [125, 126]. Some medications for epilepsy (clonazepam, felbamate, lamotrigine, oxcarbazepine, and phenobarbital) can worsen sleep [127]. Stimulants that are used to treat ADHD often have insomnia as a side-effect and, if given too late in the day, they can interfere with sleep-onset latency.

### Novel methods of assessment of sleep problems

Polysomnography (PSG) is considered the gold standard in sleep research [8], but is resource and cost intensive. PSG is also limited as an outcome measure by the foreign and stressful environment of a sleep lab, the need for specialized personnel, and short interval of assessment. As such, current strategies for assessing sleep as an outcome in clinical trials of NDDs are often limited to caregiver completed questionnaires [8] and sleep diaries [128, 129]. While these strategies are widely used for studying sleep in a variety of neurodevelopmental disorders, they rely on caregiver reporting and thus are subject to multiple forms of bias including recall as well as observer bias. For example, the time of sleep onset may be mis-reported in a sleep diary if a child’s eye-closure and silence while still awake is misinterpreted as being asleep. Night-wakings [3] could be easily missed when relying on sleep questionnaires and/or diaries. In addition, self-reporting of sleep problems is not possible in many NDDs, given the severe communication impairment. There are weak correlations between parental reports of sleep and PSG in some NDDs [70], and a recent study of children with DS also showed that parents cannot accurately predict when their child has apnea [130]. Thus, it is important to validate other objective methods of assessing sleep that are less invasive and burdensome, and that are cost effective. In recent years, wearable sensors have become smaller, lighter, cheaper, and less obtrusive, and are being increasingly utilized in sleep research studies because of their sensitivity and ease of use, which have made them suitable for longitudinal monitoring of patients within their home environment.

Actigraphy devices are worn on the wrist and can estimate sleep parameters by assessing movement during sleep. Actigraphy, which does not capture physiological parameters, may not be sufficient to detect sleep problems and differentiate between sleep stages in lieu of PSG in some NDDs [70, 131]. Newer generation sensors that utilize photoplethysmography (PPG), which uses an infrared light to measure the volumetric variations of blood circulation, along with actigraphy tend to be more accurate with detecting wake times [132]. Some wearable devices also have the capacity to capture oxygen saturation (SpO2), and the addition of pulse oximetry (which captures SpO2) permits an assessment of hypoxemia and more closely approximates what is captured during gold-standard PSG. These tend to be finger-worn devices (vs. wrist-worn) and could have important implications/use cases for NDDs such as RTT and DS, where apneas and OSA are more common. Some wearable devices that include PPG and also capture electrodermal activity (EDA), skin temperature, and heart rate variability are being used to study stress/behavioral responses (e.g., anxiety) in NDDs such as ASD.

Given the concordance of sleep problems, autonomic dysfunction, and behavioral difficulties across many NDDs, actigraphy devices may be quite useful, especially given the challenges associated with self-report and reliance on caregiver reporting. Since sensory processing issues are prevalent in NDDs [133], and studies have shown that sensory sensitivities can make it challenging for children to be compliant with wearable sensors [134], it is important to directly test the feasibility of use of these wearable sensors in NDD populations. Positioning could have an impact on data quality (wrist vs. finger worn), and, in some cases for example, if a participant is likely to exhibit mouthing of objects, a finger-worn device might need to be placed after the participant is already settled in bed. “Nearable” devices passively monitor participants within their home environment, and therefore do not require a participant to wear anything. An ongoing study in RTT is using a “nearbale” device to monitor respiration, sleep quality, and sleep stages although it is not yet known how measurements compare to gold-standard PSG.

Actigraphy scoring algorithms that have been developed with typical populations (e.g., Cole-Kripke, Sadeh) [135–137] have limitations when applied to those with NDDs in that they can vary across devices and might underestimate sleep onset latency and overestimate sleep duration [134, 138]. To assist, recent advances in machine learning and artificial intelligence (AI) have allowed for the development of models using both movement and physiological data from wearable sensors to predict sleep problems, differentiate sleep stages, and even to detect OSA [139, 140−142]. Although most current work is based on typically developing adults and has yet to be applied to children with NDDs, we recently demonstrated the utility of a machine-learning approach in RTT that combined actigraphy with physiological parameters to create a model that aligned well with PSG [131]. There are also remote assessment devices for OSA. A recent study examined the feasibility and accuracy of Level 2 home sleep apnea testing (HSAT) in children, adolescents, and adults with DS and found that Level 2 HSAT was well-tolerated, preferred by parents/caregivers as compared to PSG, and had good accuracy for detecting moderate-severe OSA [143]. This has yet to be tested more broadly in other NDDs.

Given the differences in aspects of sleep in the NDDs we discussed here as compared to typical populations as well as their sensory sensitivities, it is important to determine: (1) the utility of use of wearable sensors to assess both movement and physiological signals in NDDs, and (2) the degree to which these signals accurately predict sleep problems and OSA or other apnea episodes. It will be very important to take a stepwise approach for analytical validation [144] to determine the accuracy, prediction, and reliability of measurements in NDDs from wearable sensors before they can be used more broadly. Also, data sharing approaches (e.g., some companies allow open sourcing of data) across other intellectual and developmental disabilities research centers can further propel the use of wearable sensors and help determine the best use cases and parameters for analyses as well as clinical validation across NDDs. It will also be important to consider the cost per use for each wearable device and whether the device is reusable or disposable, especially since certain wearable and nearable devices are cost prohibitive for use in larger scaled studies such as clinical trials. Related to this, it is important to consider battery life for potential collection of sleep data over several days since up to seven days of data are recommended, for example, for determining total sleep time and sleep efficiency [145]. Finally, researchers should consider whether they will have access to raw data to develop novel algorithms, which is very important for the study of NDDs, as well as options for available data storage, and potential privacy concerns.

### Summary and conclusions

Sleep is very commonly disrupted in intellectual and developmental disabilities and these disruptions not only place a large burden on caregivers, but also adversely impact the quality of life of individuals with NDDs themselves. The advantage of studying sleep in single gene disorders is that it has high clinical translational value given the common methodologies that can be utilized across human and animal model studies to bridge gaps in mechanistic understanding that can lead to improved treatments and interventions. These disease models allow for the understanding of neural circuits that contribute to sleep disruption. For more commonly occurring NDDs such as DS and ASD, there is a need for refinement of animal models to better characterize the underlying pathophysiology that contributes to sleep problems. In addition, however, within clinical research studies, defining and separating subsets of individuals within each NDD with similar functional levels, similar mutations/genetic subtypes, and/or similar co-occurring medical/behavioral conditions can also lead to enhanced insights for more targeted interventions and treatment. The use of PSG, sleep diaries, and standardized sleep questionnaires will continue to have high utility in clinical research studies and in clinical trials with NDDs, however, emerging studies are also showing that wearable sensors offer a way to objectively measure sleep in NDDs in the home/naturalistic environment that reduces caregiver burden. These methods can help develop a novel data collection format and determine when PSG is needed, determine what signal parameters are most essential resulting in the best model fit vs. PSG, and whether wearable sensors could be used in future clinical trials. The use of wearable and nearable sensors could also allow for larger scaled studies which could provide additional insights into sleep problems and targeted treatments for each of these NDDs since many current studies have more limited generalizability given the relatively small sample sizes. The use of wearble devices could also result in the need for fewer PSG’s, could reduce caregiver burden, and could assist in formulating clinical interventions and tracking improvement over time.

## Data Availability

Data sharing is not applicable to this article as no datasets were generated or analyzed during the current study.
